# Supplementary material for: A comprehensive transcriptomic comparison of hepatocyte model systems improves selection of models for experimental use
Source: Commun Biol. 2022 Oct 14;5:1094. doi: 10.1038/s42003-022-04046-9 (PMC9568534; doi:10.1038/s42003-022-04046-9)
Supplement: Supplementary file 2 — Supplementary Information [file 42003_2022_4046_MOESM2_ESM.pdf]

Supplementary Information

A comprehensive transcriptomic comparison of hepatocyte model systems improves selection of models for experimental use

Arif Ibrahim Ardisasmita, Imre F. Schene, Indi P. Joore, Gautam Kok, Delilah Hendriks, Benedetta Artegiani, Michal Mokry, Edward E. S. Nieuwenhuis, & Sabine A. Fuchs

Supplementary Figures 1-9

Supplementary Note 1

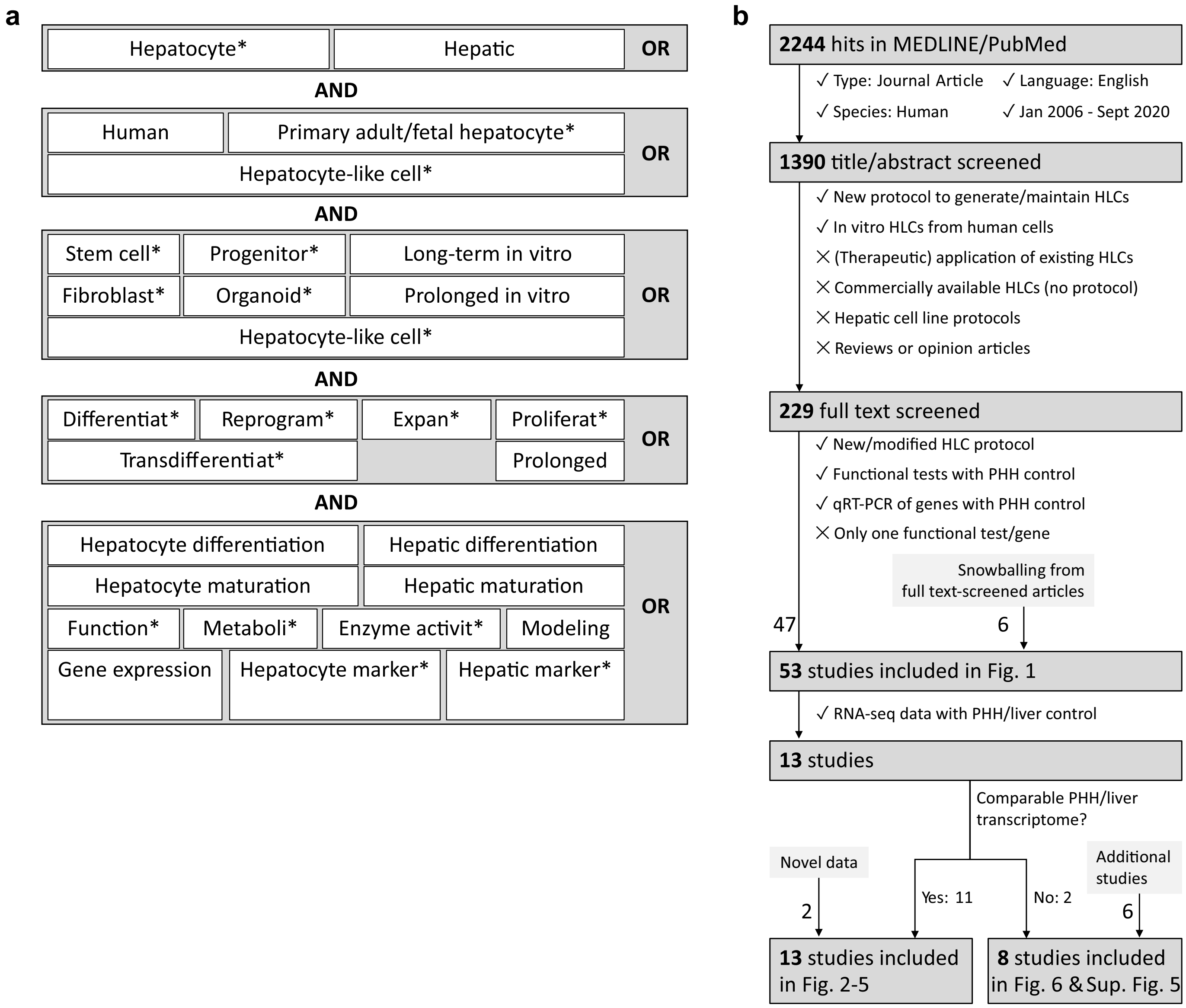

Supplementary Fig. 1. Strategy and results of systematic literature search and data collection. a Overview of terms used for systematic literature search of the PubMed database. All search terms were limited to title/abstract only using the [tiab] tag. Groups of terms, as indicated by gray boxes, were connected by 'OR' within groups and by 'AND' between groups. **b** Flow diagram describing the strategy and results of study inclusion. For each stage, inclusion and exclusion criteria are indicated by tick marks and crosses, respectively.

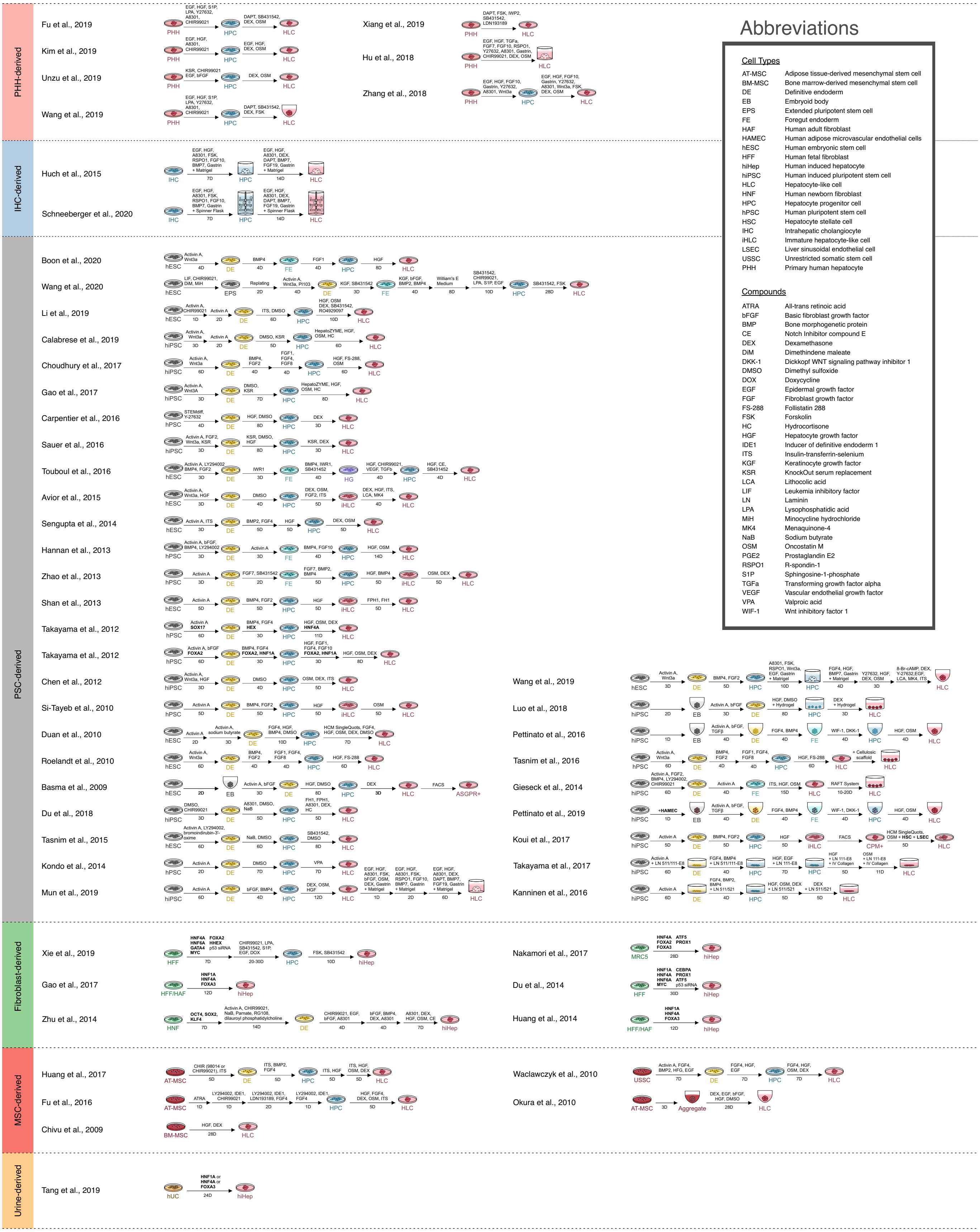

Supplementary Fig. 2. Summary of HLC generation protocols. Summary of all HLC generation protocols included in this study. Each arrow represents a (de)differentiation step; the duration of each step is presented underneath. The summay of protocols is shown concisely; for detailed descriptions please refer to each original study.

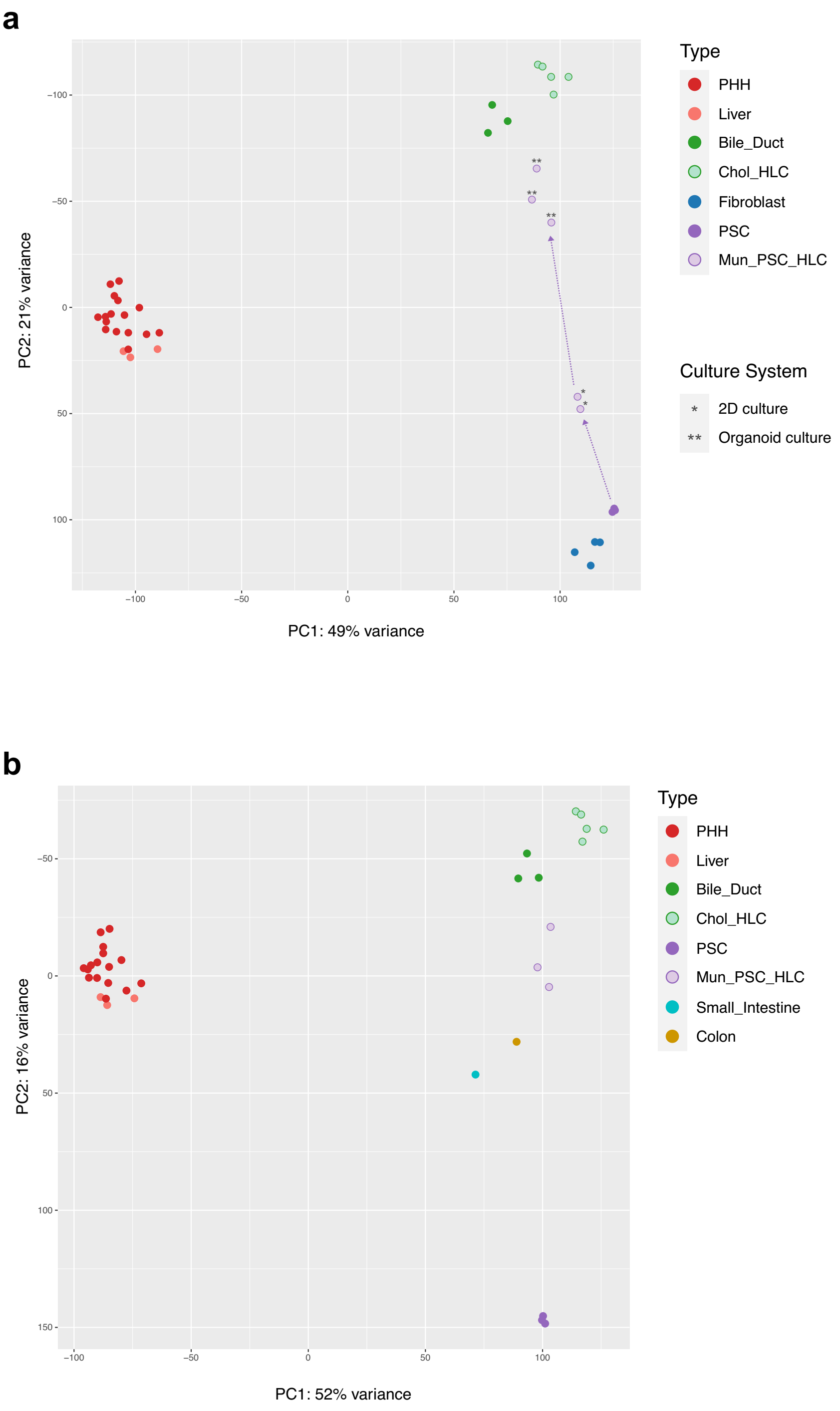

Supplementary Fig. 3. Principal component analysis of samples using/adopting Huch protocol. a Principal component analysis showing differentiation trajectory (purple arrow) of PSC-derived HLCs from Mun et al.[24] before and after adopting the organoid culture system as defined by Huch et al.[17]. **b** Principal component analysis including small intestine and colon tissue samples.

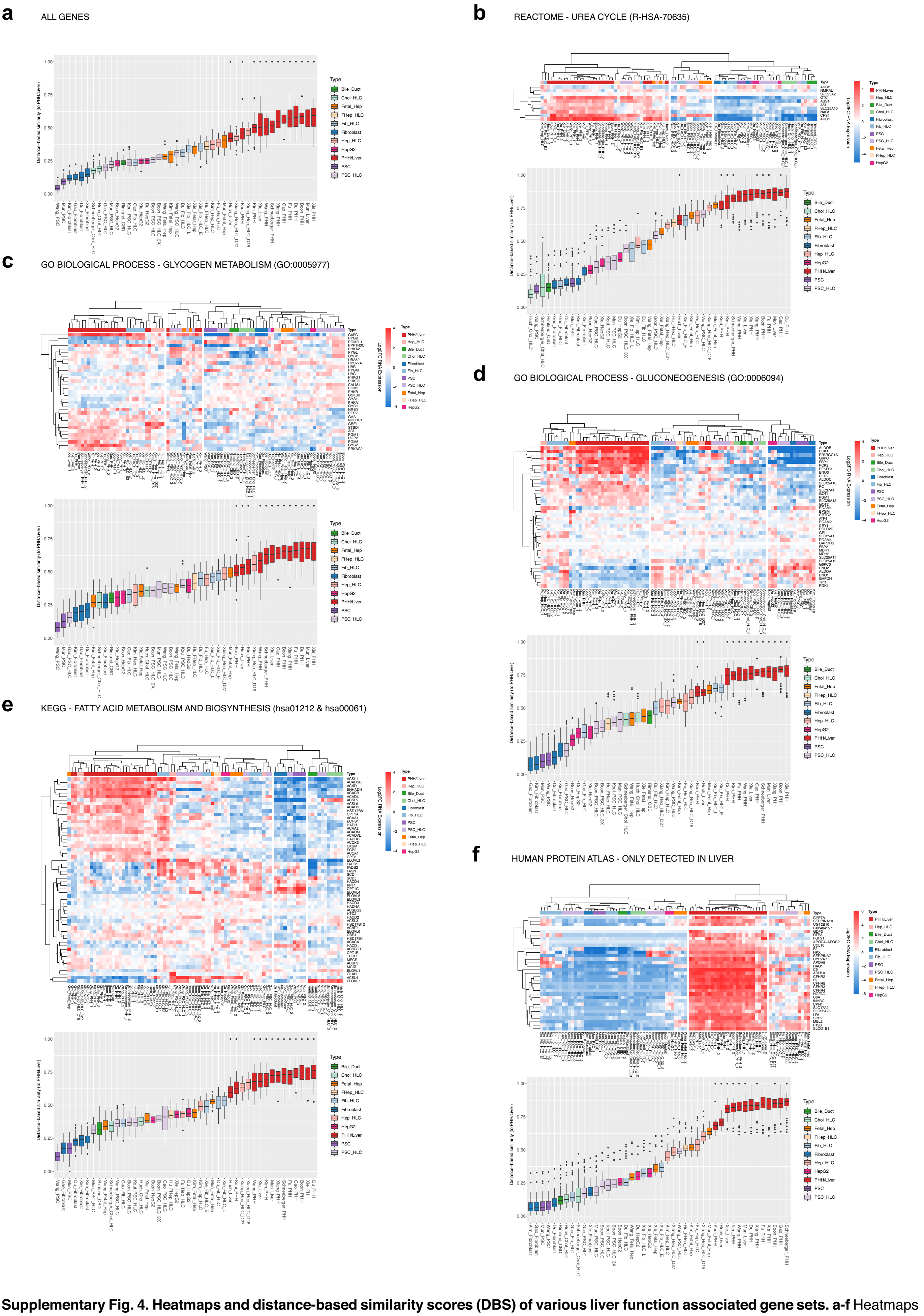

Supplementary Fig. 4. Heatmaps and distance-based similarity scores (DBS) of various liver function associated gene sets. a-f Heatmaps and DBS created using **a** all genes with total read counts ≥10 across all samples, **b** urea cycle, **c** glycogen metabolism, **d** gluconeogenesis, **e** fatty acid metabolism and biosynthesis, and **f** liver specific gene sets. Box-and-whisker plots are shown as median (line), interquartile range (box), and data range or 1.5x interquartile range (whisker).

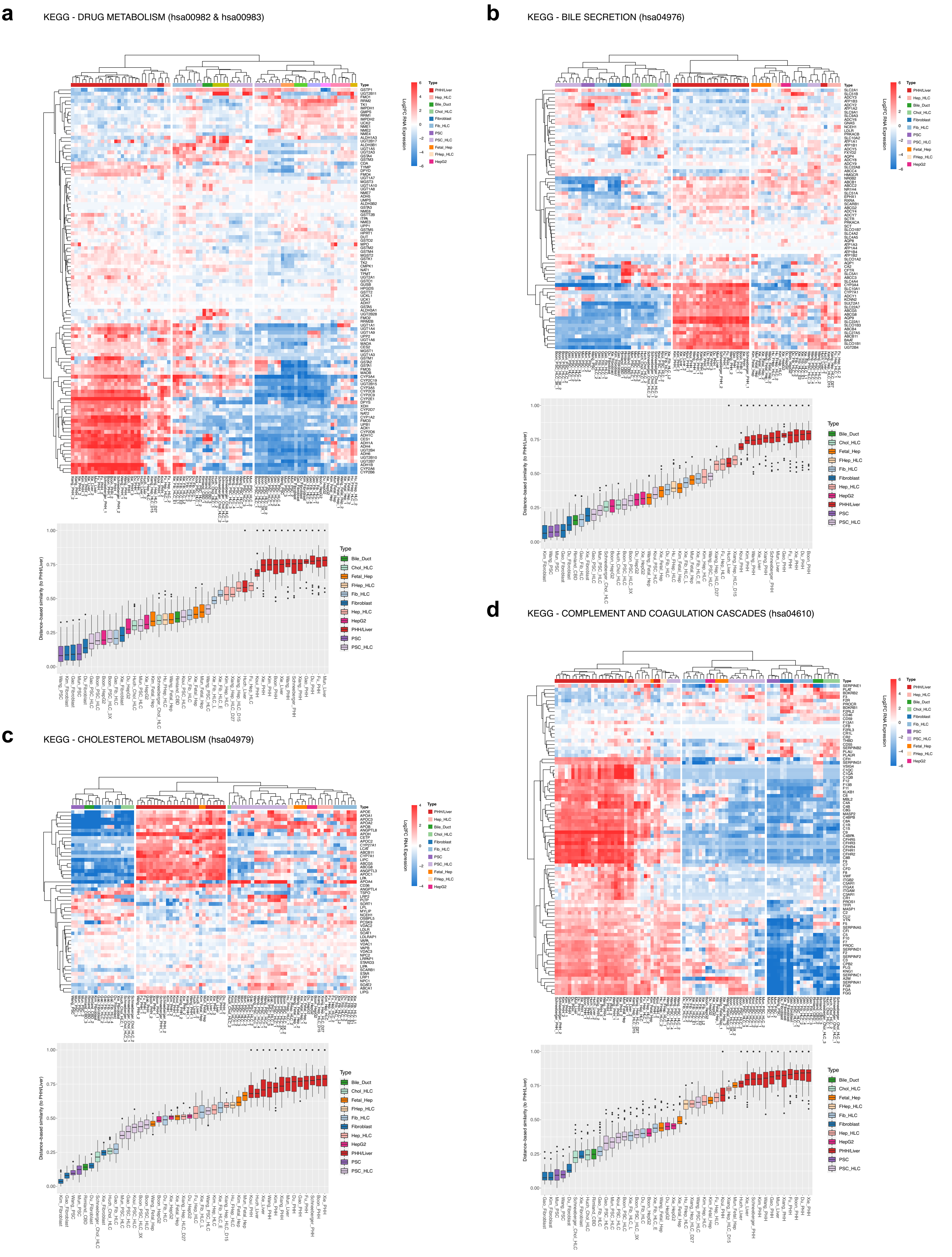

Supplementary Fig. 5. Heatmaps and distance-based similarity scores (DBS) of various liver function associated gene sets. a-d Heatmaps and DBS created using **a** drug metabolism, **b** bile secretion, **c** cholesterol metabolism, and **d** complement and coagulation cascade gene sets. Box-and-whisker plots are shown as median (line), interquartile range (box), and data range or 1.5x interquartile range (whisker).

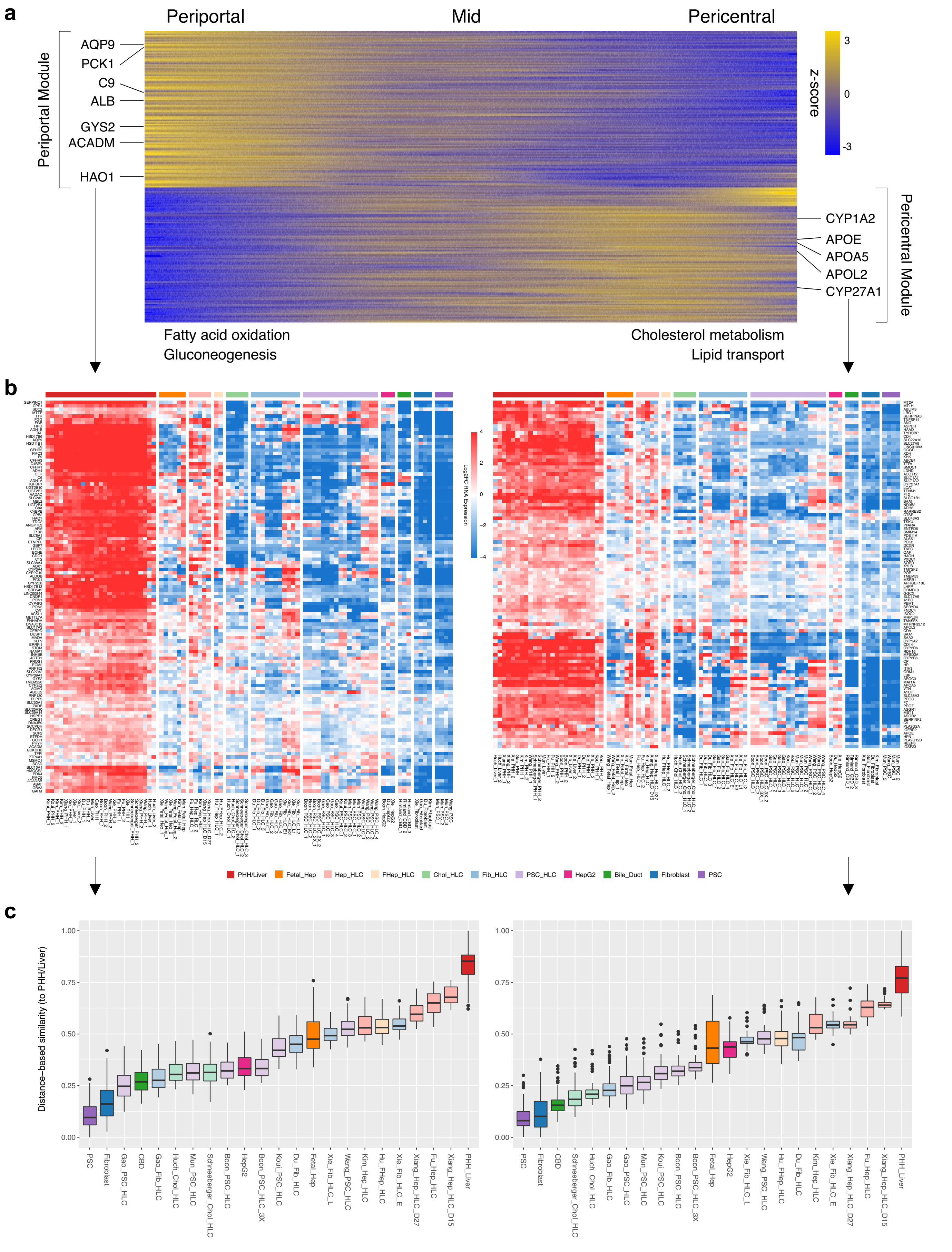

Supplementary Fig. 6. Liver zonation analysis. a Gene expression heatmap of periportal (modules 1 and 3) and pericentral (modules 33 and 34) modules in hepatocytes from the single-cell RNA sequencing data of Aizarani et al.[62]. Hepatocyte samples were ordered along the zonation axis according to the diffusion pseudo-space and self-organizing maps of Aizarani et al.[62]. **b** Heatmap showing the expression of periportal and pericentral modules in the samples included in this study. **c** DBS score of all samples using the periportal and pericentral modules. Box-and-whisker plots are shown as median (line), interquartile range (box), and data range or 1.5x interquartile range (whisker).

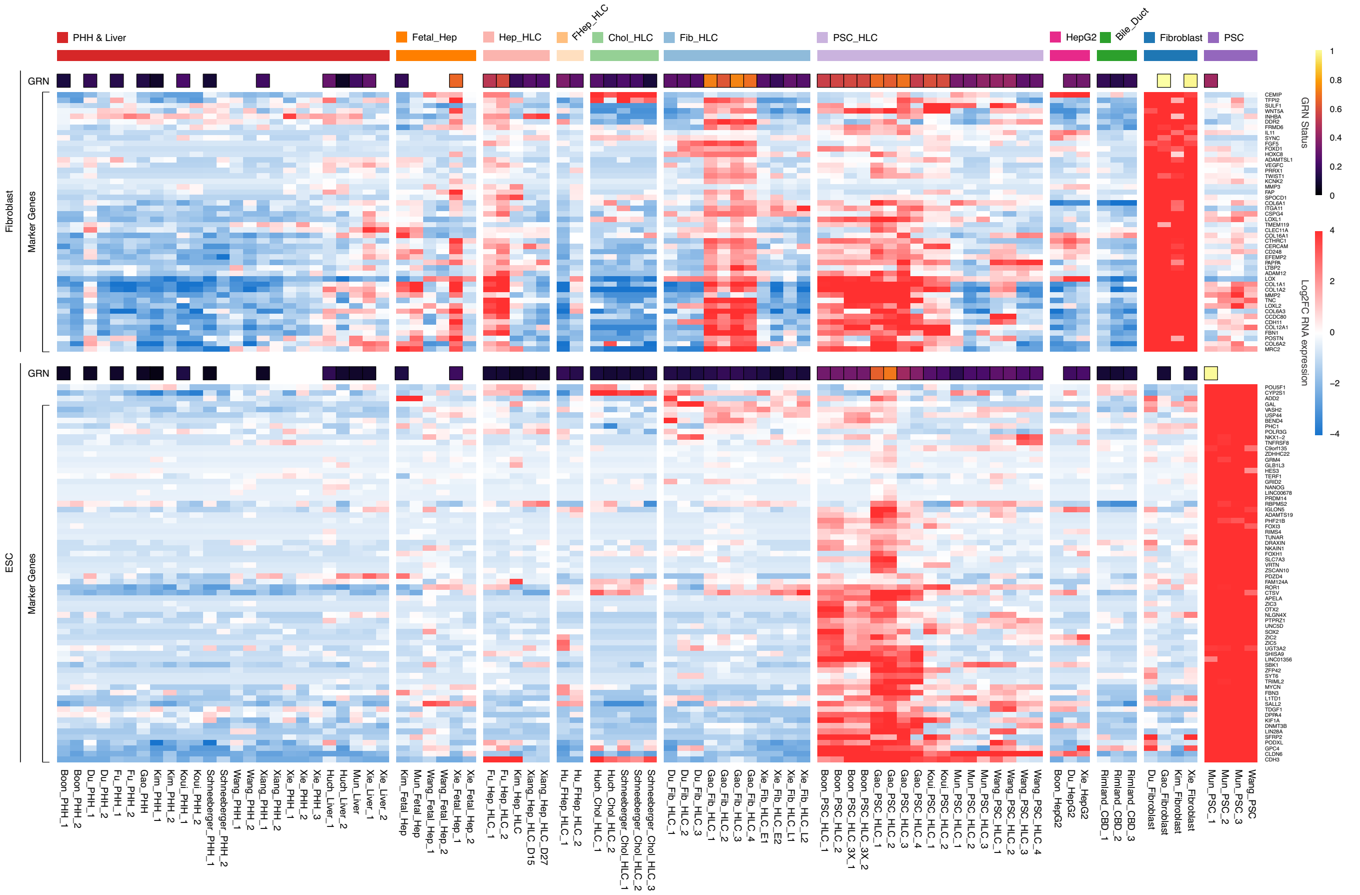

Supplementary Fig. 7. Evaluation of fibroblast and ESC identities. Heatmaps showing fibroblast and ESC gene regulatory network (GRN) status and the expression of representative genes, which make up the fibroblast and ESC GRN, in HLCs and tissue controls.

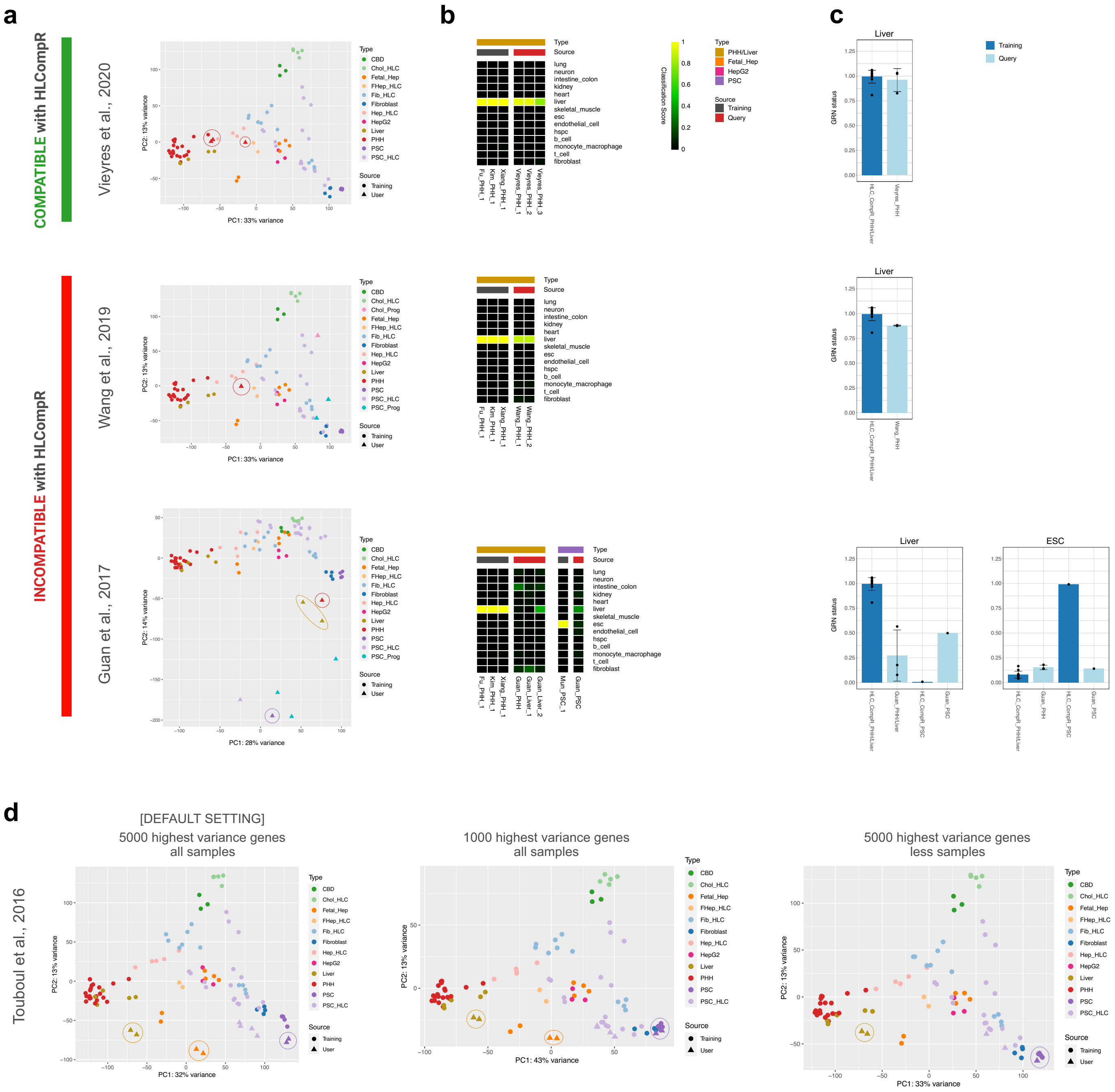

Supplementary Fig. 8. Testing the HLCompR web application using additional studies. a Principal component analysis on additional query datasets. Circles indicate the cell or tissue types in the query datasets that are also present in the training dataset (e.g., PHH, liver, and PSC). HLCompR compatibility is categorized based on the comparability of PHH and liver tissue between the query and training datasets. **b** Cell/tissue classification heatmap of representative training samples and query samples. **c** Gene regulatory network status of liver and embryonic stem cells of all PHH, liver, HepG2, and PSC samples from training dataset and query dataset. **d** Principal component analysis of Touboul *et al.*[43] query dataset using default setting of HLCompR (left) or with adjustment in the number of genes considered (middle) or number of samples included (right).

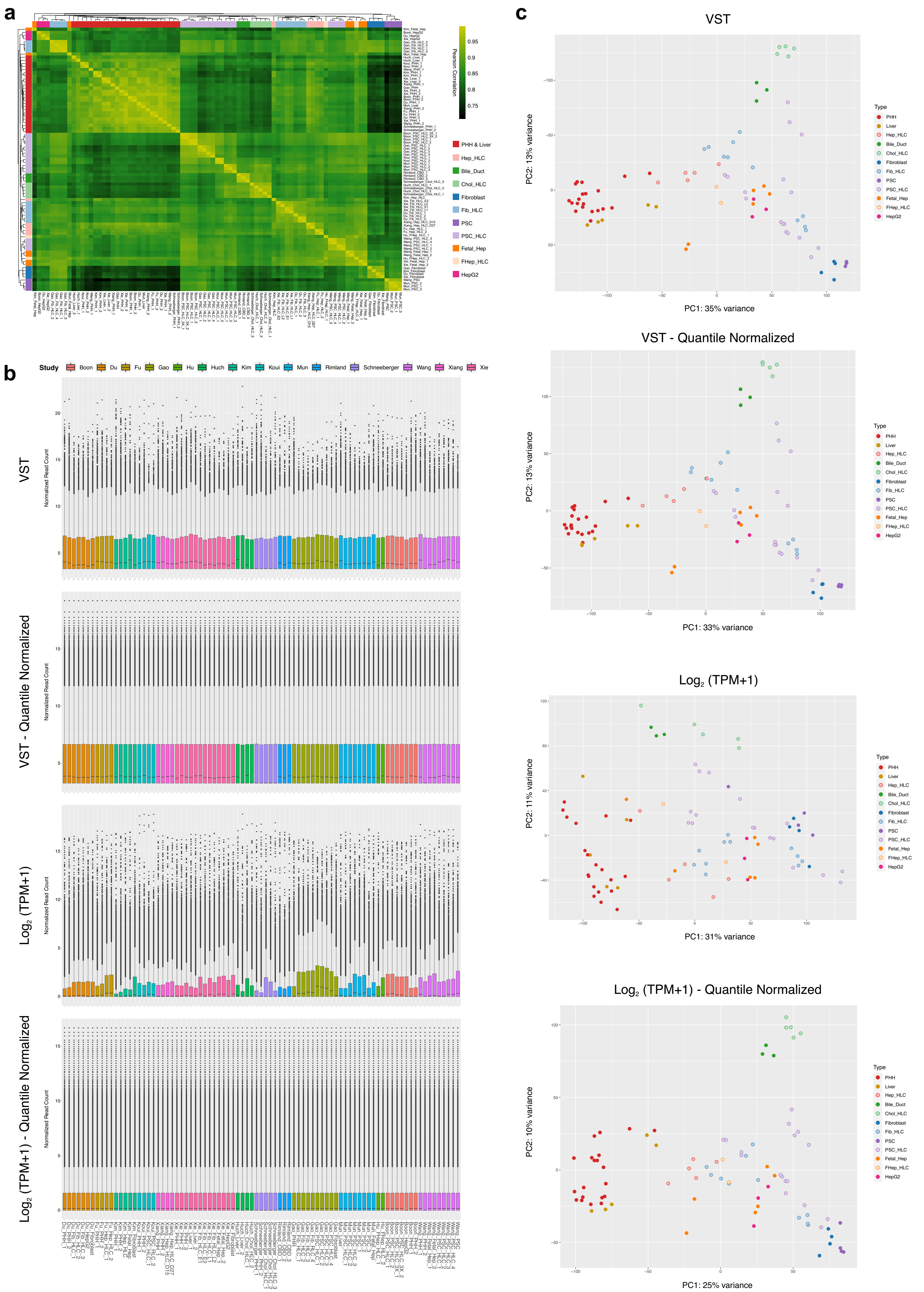

Suplementary Fig. 9. Normalization method for cross-study RNA-seq analysis. a Pearson correlation heatmap generated using all genes with total read counts ≥10 across all samples. **b** Expression levels and **c** principal component analysis of all samples using different normalization methods: variance stabilizing transformation (VST); VST followed by quantile normalization; log2 transformed transcript per million (TPM); and log2 transformed TPM followed by quantile normalization. Box-and-whisker plots are shown as median (line), interquartile range (box), and data range or 1.5x interquartile range (whisker).

Supplementary Note 1

To minimize study-specific batch effects, our approach involved uniform mapping of raw reads, variance-stabilizing transformation (VST) in DESeq2, and quantile normalization (Fig. 2a). The PCA plot using 5,000 genes with the highest variance across all samples showed that samples clustered by cell type rather than by study, suggesting no strong batch effects (Fig. 2c). This was further supported by the Pearson correlation heatmap based on the expression data of all genes, as samples clustered by cell type rather than by study (Supplementary Fig. 9a). VST and quantile normalization were crucial for this cross-study comparison, as TPM normalization resulted in non-uniform RNA expression distribution (Supplementary Fig. 9b) and suboptimal sample type clustering even after quantile normalization (Supplementary Fig. 9c).

Supplementary References

1. Fu, G. B. *et al.* Expansion and differentiation of human hepatocyte-derived liver progenitor-like cells and their use for the study of hepatotropic pathogens. *Cell Res.* **29**, 8–22 (2019).

2. Kim, Y. *et al.* Small molecule-mediated reprogramming of human hepatocytes into bipotent progenitor cells. *J. Hepatol.* **70**, 97–107 (2019).

3. Unzu, C. *et al.* Pharmacological Induction of a Progenitor State for the Efficient Expansion of Primary Human Hepatocytes. *Hepatology* **69**, 2214–2231 (2019).

4. Wang, Z. *et al.* Generation of hepatic spheroids using human hepatocyte-derived liver progenitor-like cells for hepatotoxicity screening. *Theranostics* **9**, 6690–6705 (2019).

5. Xiang, C. *et al.* Long-term functional maintenance of primary human hepatocytes in vitro. *Science (80-.).* **364**, 399–402 (2019).

6. Hu, H. *et al.* Long-Term Expansion of Functional Mouse and Human Hepatocytes as 3D Organoids. *Cell* **175**, 1591-1606.e19 (2018).

7. Zhang, K. *et al.* In Vitro Expansion of Primary Human Hepatocytes with Efficient Liver Repopulation Capacity. *Cell Stem Cell* **23**, 806-819.e4 (2018).

8. Huch, M. *et al.* Long-term culture of genome-stable bipotent stem cells from adult human liver. *Cell* **160**, 299–312 (2015).

9. Schneeberger, K. *et al.* Large-Scale Production of LGR5-Positive Bipotential Human Liver Stem Cells. *Hepatology* **72**, 257–270 (2020).

10. Boon, R. *et al.* Amino acid levels determine metabolism and CYP450 function of hepatocytes and hepatoma cell lines. *Nat. Commun.* **11**, (2020).

11. Wang, Q. *et al.* Generation of human hepatocytes from extended pluripotent stem cells. *Cell Res.* **30**, 810–813 (2020).

12. Li, Z. *et al.* Generation of qualified clinical-grade functional hepatocytes from human embryonic stem cells in chemically defined conditions. *Cell Death Dis.* **10**, (2019).

13. Calabrese, D. *et al.* Liver biopsy derived induced pluripotent stem cells provide unlimited supply for the generation of hepatocyte-like cells. *PLoS One* **14**, 1–27 (2019).

14. Choudhury, Y. *et al.* Patient-specific hepatocyte-like cells derived from induced pluripotent stem cells model pazopanib-mediated hepatotoxicity. *Sci. Rep.* **7**, (2017).

15. Gao, Y. *et al.* Distinct Gene Expression and Epigenetic Signatures in Hepatocyte-like Cells Produced by Different Strategies from the Same Donor. *Stem Cell Reports* **9**, 1813–1824 (2017).

16. Carpentier, A. *et al.* Hepatic differentiation of human pluripotent stem cells in miniaturized format suitable for high-throughput screen. *Stem Cell Res.* **16**, 640–650 (2016).

17. Sauer, V. *et al.* Human urinary epithelial cells as a source of engraftable hepatocyte-like cells using stem cell technology. *Cell Transplant.* **25**, 2221–2243 (2016).

18. Touboul, T. *et al.* Stage-specific regulation of the WNT/β-catenin pathway enhances differentiation of hESCs into hepatocytes. *J. Hepatol.* **64**, 1315–1326 (2016).

19. Avior, Y. *et al.* Microbial-Derived Lithocholic Acid and Vitamin K2 Drive the Metabolic Maturation of Pluripotent Stem Cells-Derived and Fetal Hepatocytes. *Hepatology* **62**, 265–

278 (2015).

20. Sengupta, S. *et al.* Aggregate culture of human embryonic stem cell-derived hepatocytes in suspension are an improved In Vitro model for drug metabolism and toxicity testing. *Toxicol. Sci.* **140**, 236–245 (2014).

21. Hannan, N. R. F., Segeritz, C. P., Touboul, T. & Vallier, L. Production of hepatocyte-like cells from human pluripotent stem cells. *Nat. Protoc.* **8**, 430–437 (2013).

22. Zhao, D. *et al.* Promotion of the efficient metabolic maturation of human pluripotent stem cell-derived hepatocytes by correcting specification defects. *Cell Res.* **23**, 157–161 (2013).

23. Shan, J. *et al.* Identification of small molecules for human hepatocyte expansion and iPS differentiation. *Nat. Chem. Biol.* **9**, 514–520 (2013).

24. Takayama, K. *et al.* Generation of metabolically functioning hepatocytes from human pluripotent stem cells by FOXA2 and HNF1α transduction. *J. Hepatol.* **57**, 628–636 (2012).

25. Takayama, K. *et al.* Efficient generation of functional hepatocytes from human embryonic stem cells and induced pluripotent stem cells by HNF4α transduction. *Mol. Ther.* **20**, 127–137 (2012).

26. Chen, Y. F. *et al.* Rapid generation of mature hepatocyte-like cells from human induced pluripotent stem cells by an efficient three-step protocol. *Hepatology* **55**, 1193–1203 (2012).

27. Si-Tayeb, K. *et al.* Highly efficient generation of human hepatocyte-like cells from induced pluripotent stem cells. *Hepatology* **51**, 297–305 (2010).

28. Duan, Y. *et al.* Differentiation and characterization of metabolically functioning hepatocytes from human embryonic stem cells. *Stem Cells* **28**, 674–686 (2010).

29. Roelandt, P. *et al.* Human embryonic and rat adult stem cells with primitive endoderm-like phenotype can be fated to definitive endoderm, and finally hepatocyte-like cells. *PLoS One* **5**, (2010).

30. Basma, H. *et al.* Differentiation and Transplantation of Human Embryonic Stem Cell-Derived Hepatocytes. *Gastroenterology* **136**, 990-999.e4 (2009).

31. Du, C. *et al.* Highly efficient and expedited hepatic differentiation from human pluripotent stem cells by pure small-molecule cocktails. *Stem Cell Res. Ther.* **9**, (2018).

32. Tasnim, F., Phan, D., Toh, Y. C. & Yu, H. Cost-effective differentiation of hepatocyte-like cells from human pluripotent stem cells using small molecules. *Biomaterials* **70**, 115–125 (2015).

33. Kondo, Y. *et al.* Histone deacetylase inhibitor valproic acid promotes the differentiation of human induced pluripotent stem cells into hepatocyte-like cells. *PLoS One* **9**, 1–11 (2014).

34. Mun, S. J. *et al.* Generation of expandable human pluripotent stem cell-derived hepatocyte-like liver organoids. *J. Hepatol.* **71**, 970–985 (2019).

35. Wang, S. *et al.* Human ESC-derived expandable hepatic organoids enable therapeutic liver repopulation and pathophysiological modeling of alcoholic liver injury. *Cell Res.* **29**, 1009–1026 (2019).

36. Luo, Y. *et al.* Three-dimensional hydrogel culture conditions promote the differentiation of human induced pluripotent stem cells into hepatocytes. *Cytotherapy* **20**, 95–107 (2018).

37. Pettinato, G. *et al.* Scalable Differentiation of Human iPSCs in a Multicellular Spheroid-based 3D Culture into Hepatocyte-like Cells through Direct Wnt/β-catenin Pathway Inhibition. *Sci.*

Rep. **6**, 1–17 (2016).

38. Tasnim, F. *et al.* Functionally Enhanced Human Stem Cell Derived Hepatocytes in Galactosylated Cellulosic Sponges for Hepatotoxicity Testing. *Mol. Pharm.* **13**, 1947–1957 (2016).

39. Gieseck, R. L. *et al.* Maturation of induced pluripotent stem cell derived hepatocytes by 3D-culture. *PLoS One* **9**, (2014).

40. Pettinato, G. *et al.* Generation of fully functional hepatocyte-like organoids from human induced pluripotent stem cells mixed with Endothelial Cells. *Sci. Rep.* **9**, 1–21 (2019).

41. Koui, Y. *et al.* An In Vitro Human Liver Model by iPSC-Derived Parenchymal and Non-parenchymal Cells. *Stem Cell Reports* **9**, 490–498 (2017).

42. Takayama, K. *et al.* Generation of safe and therapeutically effective human induced pluripotent stem cell-derived hepatocyte-like cells for regenerative medicine. *Hepatol. Commun.* **1**, 1058–1069 (2017).

43. Kanninen, L. K. *et al.* Laminin-511 and laminin-521-based matrices for efficient hepatic specification of human pluripotent stem cells. *Biomaterials* **103**, 86–100 (2016).

44. Xie, B. *et al.* A two-step lineage reprogramming strategy to generate functionally competent human hepatocytes from fibroblasts. *Cell Res.* **29**, 696–710 (2019).

45. Zhu, S. *et al.* Mouse liver repopulation with hepatocytes generated from human fibroblasts. *Nature* **508**, 93–97 (2014).

46. Nakamori, D., Akamine, H., Takayama, K., Sakurai, F. & Mizuguchi, H. Direct conversion of human fibroblasts into hepatocyte-like cells by ATF5, PROX1, FOXA2, FOXA3, and HNF4A transduction. *Sci. Rep.* **7**, (2017).

47. Du, Y. *et al.* Human hepatocytes with drug metabolic function induced from fibroblasts by lineage reprogramming. *Cell Stem Cell* **14**, 394–403 (2014).

48. Huang, P. *et al.* Direct reprogramming of human fibroblasts to functional and expandable hepatocytes. *Cell Stem Cell* **14**, 370–384 (2014).

49. Huang, J., Guo, X., Li, W. & Zhang, H. Activation of Wnt/β-catenin signalling via GSK3 inhibitors direct differentiation of human adipose stem cells into functional hepatocytes. *Sci. Rep.* **7**, 1–12 (2017).

50. Fu, Y. *et al.* Rapid generation of functional hepatocyte-like cells from human adipose-derived stem cells. *Stem Cell Res. Ther.* **7**, 1–12 (2016).

51. Chivu, M. *et al.* In vitro hepatic differentiation of human bone marrow mesenchymal stem cells under differential exposure to liver-specific factors. *Transl. Res.* **154**, 122–132 (2009).

52. Waclawczyk, S., Buchheiser, A., Flögel, U., Radke, T. F. & Kögler, G. In vitro differentiation of unrestricted somatic stem cells into functional hepatic-like cells displaying a hepatocyte-like glucose metabolism. *J. Cell. Physiol.* **225**, 545–554 (2010).

53. Okura, H. *et al.* Properties of hepatocyte-like cell clusters from human adipose tissue-derived mesenchymal stem cells. *Tissue Eng. - Part C Methods* **16**, 761–770 (2010).

54. Tang, W. *et al.* Chemical cocktails enable hepatic reprogramming of human urine-derived cells with a single transcription factor. *Acta Pharmacol. Sin.* **40**, 620–629 (2019).
