# Supplementary material for: A comprehensive transcriptomic comparison of hepatocyte model systems improves selection of models for experimental use
Source: Commun Biol. 2022 Oct 14;5:1094. doi: 10.1038/s42003-022-04046-9 (PMC9568534; doi:10.1038/s42003-022-04046-9)
Supplement: Supplementary file 3 — Description of Additional Supplementary Data [file 42003_2022_4046_MOESM3_ESM.docx]

**Description of Additional Supplementary Files**

**File name:** Supplementary Data 1

**Description:** Summary table of reported functional assays and gene expression

**File name:** Supplementary Data 2

**Description:** Detailed information on RNA-seq data used in the study

**File name:** Supplementary Data 3

**Description:** Reported functional and expression data

**File name:** Supplementary Data 4

**Description:** Count matrices of the RNA-seq data

**File name:** Supplementary Data 5

**Description:** Median DBS based on hepatocyte functions and liver zonation gene sets

**File name:** Supplementary Data 6

**Description:** HLC identity analysis

**File name:** Supplementary Data 7

**Description:** Validation of transcriptomic

**File name:** Supplementary Data 8

**Description:** Additional HLC samples
