# Supplementary material for: A comprehensive transcriptomic comparison of hepatocyte model systems improves selection of models for experimental use
Source: Commun Biol. 2022 Oct 14;5:1094. doi: 10.1038/s42003-022-04046-9 (PMC9568534; doi:10.1038/s42003-022-04046-9)
Supplement: Supplementary file 12 — Reporting Summary-New [file 42003_2022_4046_MOESM12_ESM.pdf]

nature research

Corresponding author(s): Sabine A. Fuchs

Last updated by author(s): Sep 27, 2022

# Reporting Summary

Nature Research wishes to improve the reproducibility of the work that we publish. This form provides structure for consistency and transparency in reporting. For further information on Nature Research policies, see our Editorial Policies and the Editorial Policy Checklist.

## Statistics

For all statistical analyses, confirm that the following items are present in the figure legend, table legend, main text, or Methods section.

| n/a | Confirmed | |
|---|---|---|
| ☐ | ☒ | The exact sample size (*n*) for each experimental group/condition, given as a discrete number and unit of measurement |
| ☐ | ☒ | A statement on whether measurements were taken from distinct samples or whether the same sample was measured repeatedly |
| ☐ | ☒ | The statistical test(s) used AND whether they are one- or two-sided<br>*Only common tests should be described solely by name; describe more complex techniques in the Methods section.* |
| ☐ | ☒ | A description of all covariates tested |
| ☐ | ☒ | A description of any assumptions or corrections, such as tests of normality and adjustment for multiple comparisons |
| ☐ | ☒ | A full description of the statistical parameters including central tendency (e.g. means) or other basic estimates (e.g. regression coefficient) AND variation (e.g. standard deviation) or associated estimates of uncertainty (e.g. confidence intervals) |
| ☐ | ☒ | For null hypothesis testing, the test statistic (e.g. *F*, *t*, *r*) with confidence intervals, effect sizes, degrees of freedom and *P* value noted<br>*Give P values as exact values whenever suitable.* |
| ☒ | ☐ | For Bayesian analysis, information on the choice of priors and Markov chain Monte Carlo settings |
| ☒ | ☐ | For hierarchical and complex designs, identification of the appropriate level for tests and full reporting of outcomes |
| ☐ | ☒ | Estimates of effect sizes (e.g. Cohen's *d*, Pearson's *r*), indicating how they were calculated |

*Our web collection on statistics for biologists contains articles on many of the points above.*

## Software and code

Policy information about availability of computer code

| | |
|---|---|
| Data collection | The PubMed database was systematically searched for studies that mention the development or evaluation of HLC culturing protocols. Raw reads from the RNA-seq data were obtained from the European Nucleotide Archive (ENA, https://www.ebi.ac.uk/ena). |
| Data analysis | Raw reads were processed using Galaxy (https://usegalaxy.eu/) web-based platform (Afgan et al., 2018). Sample quality was assessed using FastQC tool (Galaxy Version 0.72). Low quality reads and adapter sequences were trimmed using Cutadapt (Galaxy Version 1.66.6). Alignment of the raw reads and quantification of gene expression were performed using RNA STAR tool (Galaxy Version 2.7.2b). Reads were mapped to Gencode human reference genome sequence release 33 (GRCh38.p13) and Gencode comprehensive gene annotation v33, using default parameters. Read counts were obtained using the "--quantMode GeneCounts" option in the RNA STAR tool. The script for analysis of read counts in the HLCompR application is available in https://github.com/iardisasmita/HLCompR. In brief, normalized counts were obtained by applying the DESeq2 variance-stabilizing transformation (VST) to the read counts using the 'DESeq2' R package (Love et al., 2014) followed by quantile normalization using the 'preprocessCore' R package (Bolstad et al., 2003). |

For manuscripts utilizing custom algorithms or software that are central to the research but not yet described in published literature, software must be made available to editors and reviewers. We strongly encourage code deposition in a community repository (e.g. GitHub). See the Nature Research guidelines for submitting code & software for further information.

## Data

Policy information about availability of data

All manuscripts must include a data availability statement. This statement should provide the following information, where applicable:

- Accession codes, unique identifiers, or web links for publicly available datasets
- A list of figures that have associated raw data
- A description of any restrictions on data availability

Summary of previously published reported assays and gene expression data included in this study is available in Supplementary Data 1. List of RNA-sequencing data used in this study is available in Supplementary Data 2. Raw counts, normalized counts, and metadata of the RNA-sequencing data used in this study is available in Supplementary Data 4. Source data underlying the figures are available in Supplementary Data 3-8. Processed RNA-sequencing data generated in this study have been deposited in the NCBI Gene Expression Omnibus database under accession number GSE214097. Raw RNA-sequencing data are not publicly available due to potential information that could compromise donor consent. All materials supporting the findings of this study are available from the corresponding author upon reasonable request.

# Field-specific reporting

Please select the one below that is the best fit for your research. If you are not sure, read the appropriate sections before making your selection.

☒ Life sciences ☐ Behavioural & social sciences ☐ Ecological, evolutionary & environmental sciences

For a reference copy of the document with all sections, see nature.com/documents/nr-reporting-summary-flat.pdf

# Life sciences study design

All studies must disclose on these points even when the disclosure is negative.

| | |
|---|---|
| Sample size | No sample-size calculation was performed. For transcriptomic studies we included a minimum of two samples per type of hepatocyte-like cell (HLC) model, if available. In experiments designed to compare the functional capabilities of HLCs, we included two biological replicates for each HLC model. |
| Data exclusions | No data was excluded from analysis. |
| Replication | No attempts at replication of experiments in the paper failed. Data in this paper was reproducible. |
| Randomization | Not applicable. All functional tests were performed in all available HLC models in our lab. No specific wells were selected for inclusion in the vairous functional assays. |
| Blinding | The analysis of functional assays was performed by automated colorimetric and luminescent read outs, which are not subject to human bias. Therefore, blinding was not relevant for these analyses. |

# Reporting for specific materials, systems and methods

We require information from authors about some types of materials, experimental systems and methods used in many studies. Here, indicate whether each material, system or method listed is relevant to your study. If you are not sure if a list item applies to your research, read the appropriate section before selecting a response.

### Materials & experimental systems

| n/a | Involved in the study | |
|---|---|---|
| ☒ | ☐ | Antibodies |
| ☐ | ☒ | Eukaryotic cell lines |
| ☒ | ☐ | Palaeontology and archaeology |
| ☒ | ☐ | Animals and other organisms |
| ☐ | ☒ | Human research participants |
| ☒ | ☐ | Clinical data |
| ☒ | ☐ | Dual use research of concern |

### Methods

| n/a | Involved in the study | |
|---|---|---|
| ☒ | ☐ | ChIP-seq |
| ☒ | ☐ | Flow cytometry |
| ☒ | ☐ | MRI-based neuroimaging |

## Eukaryotic cell lines

Policy information about cell lines

| | |
|---|---|
| Cell line source(s) | HepG2 (ATCC) |
| Authentication | Cells were authenticated by the supplier using STR analysis |

| | |
|---|---|
| Mycoplasma contamination | All cell lines tested negative in repeated (3-monthly) mycoplasma contamination tests. |
| Commonly misidentified lines (See ICLAC register) | None used. |

## Human research participants

Policy information about studies involving human research participants

| | |
|---|---|
| Population characteristics | Tissue biopsies from livers of healthy donors were obtained during surgery in the Erasmus MC, Rotterdam. Human fetal livers were obtained from Leiden University Medical Centre (MC). All patient materials were used after written informed consent. |
| Recruitment | Tissue biopsies from livers of healthy donors were obtained during surgery in the Erasmus MC, Rotterdam. Human fetal livers were obtained from Leiden University Medical Centre (MC). All patient materials were used after written informed consent. |
| Ethics oversight | The study was approved by the responsible local ethics committees (Institutional Review Board of the University Medical Center Utrecht (STEM: 10-402/K; TcBio 14-008; Metabolic Biobank: 19-489), Erasmus MC Medical Ethical Committee (MEC-2014-060), and the Dutch Ethical Medical Council (Leiden University MC)). |

Note that full information on the approval of the study protocol must also be provided in the manuscript.
